# Elevated CO_2_ alters transgene methylation not only in promoterregion but also in codingregion of *Bt* rice under different N-fertilizer levels

**DOI:** 10.1038/s41598-020-75121-6

**Published:** 2020-10-23

**Authors:** Yanmin Liu, Yanhui Wang, Geng Chen, Chunxu Li, Shoulin Jiang, Megha N. Parajulee, Fajun Chen

**Affiliations:** 1 Department of Entomology, College of Plant Protection, Nanjing Agricultural University, Nanjing, 210095 China; 2 Personnel Department, Qingdao Agricultural University, Qingdao, 266109 China; 3 Texas A&M AgriLife Research and Extension Center, Lubbock, TX 79403 USA

**Keywords:** Biological techniques, Plant sciences

## Abstract

The earth has been undergoing climate change, especially in recent years, driven by increasing concentration of atmospheric carbon dioxide (CO_2_) and rising earth-surface temperature, which could reduce N allocation to Bt toxin for transgenic *Bt* crops (*Bt* crops), but the N fertilization is considered to be an effective method to enhance the C–N balance in *Bt* crops in the case of elevated CO_2_ in future. DNA methylation not only in promoterregion but also in codingregion of transgene plays a critical role in transgene expression regulation and silencing of transgenic crops. Recent research has emphasized the risks of increased transgene silencing of *Bacillus thuringiensis* (*Bt*) rice under elevated CO_2_. In this study, the effects of elevated CO_2_ (vs. ambient CO_2_) on exogenous *Bt* toxins and transgene expression in promoterregion and codingregion of *Bt* rice during tillering stage (cv. HH1 expressing fused *Cry1Ab/Cry1Ac*) were evaluated under three nitrogen (N) fertilizer rate (1/4, 1 and 2 N levels). The aboveground and belowground biomass, and foliar *Bt* protein content of *Bt* rice were all significantly increased with the augmentation of N-fertilizer. And elevated CO_2_ significantly increased belowground biomass, total soluble protein content, transgene methylation levels in promoterregion (P1), and in total of promoterregion(P1) and codingregion (P2 + P3) (i.e., P1 + P2 + P3) at 1 N level, and it also increased transgene methylation levels in codingregion (P2), and in total of promoterregion and codingregion (P1 + P2 + P3) at 2 N level. In addition, elevated CO_2_ decreased foliar Bt protein content at 1 N level. The transgene methylation levels in promoterregion and codingregion were negatively correlated with *Bt*-transgene expression level. The methylation level of cytosines located at CG sites was higher than those at CHG and CHH sites in P1, P2 and P3 fragments regardless of the CO_2_ or N-fertilizer level. The correlation of transgene mehtylation in promoterregion with transgene expression is even stronger than that in codingregion. These data indicate that N fertilization supply will increase the Bt toxin content in transgenic *Bt* rice, especially under elevated CO_2_.

## Introduction

Global atmospheric carbon dioxide (CO_2_) concentration has increased from 280 ppm in pre-industrial to 404 ppm currently^1^. It has been projected that it will grow up to 700 ppm at the end of this century^2^. Elevated CO_2_ can increase photosynthetic rate, biomass, and C:N ratio of plants^3–6^. Plants grown under elevated CO_2_ accumulate increased level of nonstructural carbohydrates and afford lower nutritional quality of plant tissues for herbivorous insect pests^7^. Broadly speaking, assimilation and allocation profiles of carbon and nitrogen in plant under elevated CO_2_ will change the primary and secondary metabolites of plants, thereby affecting the aboveground and belowground herbivorous insects^8–10^.

Rice (*Oryza sativa* L.) is a stable food for more than half of the world’s population^11^. Unfortunately, rice yields suffer huge losses by insect pests especially lepidopteran pests^12^. Researchers have developed transgenic rice varieties that produce insecticidal Cry toxins from *Bacillus thuringiensis* (*Bt*) in order to control target lepidopteran pests^13,14^. Among them, Huahui 1 (HH1), expressing the fused *Cry1Ab/1Ac* gene, has high resistance to the target lepidopteran pests of rice and has been issued bio-safety certificates in China^15^.

Because a biologically effective exogenous insect-resistant Bt toxin is expressed in transgenic rice, the stability of Bt toxin expression in plant tissues of *Bt* rice under elevated CO_2_ has been of great interests among researchers. Previous studies have investigated the effects of elevated CO_2_ on performance of *Bt* crops and stability of the transgenic traits^16–19^. Some studies have suggested that the exogenous gene expression in *Bt* plants transfers certain nutrients from the normal physiological pathways which may change the C-N balance, especially in the case of changed abiotic conditions^16–18,20^. The application of N fertilization can alleviate such nutrient diversion^21^. Coviella et al. found that elevated CO_2_ reduced N allocation to Bt toxin, but the reduction was largely diverted by the augmentation of nitrogen^22^. Hence, the N fertilization is considered to be an effective method to enhance the C–N balance in *Bt* plants in the case of elevated CO_2_ in the future^16,22^.

DNA methylation represents a stable epigenetic mechanism in regulating gene expression^23–26^. Numerous studies have proven that DNA methylation plays a critical role on many aspects of plant growth, including flower development, responses to environment stress, transgene expression regulation and silencing^27–31^. Transgene silencing mainly includes two forms, that is, transcriptional gene silencing (TGS), in which DNA methylation occurs in promoter-region, and posttranscriptional gene silencing (PTGS), in which DNA methylation occurs in coding sequences^32–34^. Li et al. reported that promoter-region methylation repressed gene expression and coding-region methylation usually positively associated with gene expression^35^. During seedling stage of *Bt* rice, the foliar coding-region methylation keeped at low level and showed a moderate regulation of *Bt* gene expression under elevated CO_2_ and N augmentation situation^19^. However, how did promoter-region methylation regulate the *Bt*-transgene expression of *Bt* rice under elevated CO_2_ was still unclear. Tillering stage is a key period for the construction of rice population. The number of tillers and the quality of growth determine the formation of final yield. So, the higher foliar exogenous-toxin protein content of *Bt* rice grown under elevated CO_2_ is important to control target lepidopteran pests and thus get higher yields. Investigating how transgene methylation in promoterregion and codingregion regulate the exogenous transgene expression under elevated CO_2_ is vital to ensure higher *Bt*-transgene expression level for *Bt* rice.

In this study, the effects of elevated CO_2_ on *Bt*-transgene expression in promoterregion and codingregion of *Bt* rice during tillering stage were investigate under different N-fertilizer levels. The aims of this study were to: (1) explore whether N-fertilizer application under elevated CO_2_ condition can alleviate or eliminate the nitrogen limitation in *Bt* rice, (2) investigate how the transgene methylation levels in promoterregion and codingregion regulates *Bt*-transgene expression under elevated CO_2_ condition.

## Results

### Belowground and aboveground biomass of Bt rice

**Figure 1:**
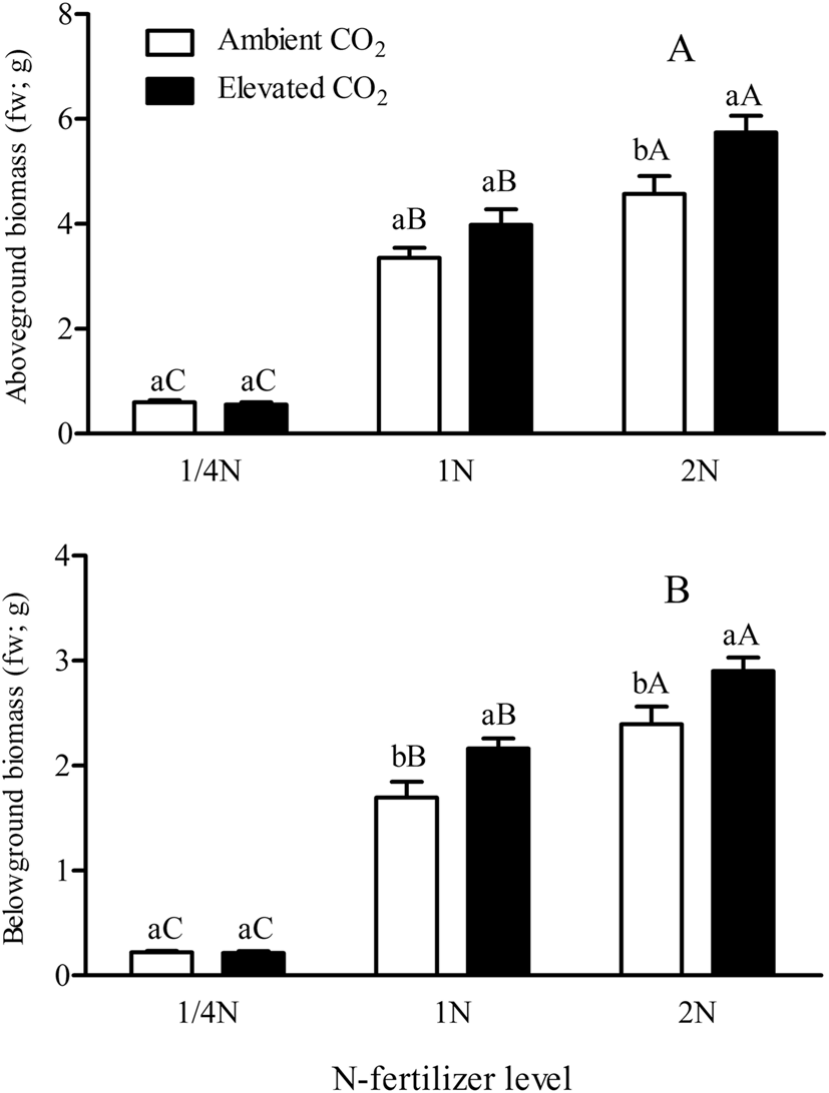
Aboveground (**A**) and belowground (**B**) biomass of *Bt* rice with fused *Cry1Ab/Ac* during tillering stage, grown under ambient and elevated CO_2_ with different N-fertilizer levels. (Values are mean ± SE. Values denoted by different lowercase and uppercase letters indicate significant differences between the ambient CO_2_ and elevated CO_2_ for same N-fertilizer rates, and between the different N-fertilizer rates for same CO_2_ level by LSD test at *P* < 0.05. The same in Figs. 2, 3, 4, 5, 6, 7).

CO_2_, N-fertilizer levels and their interaction were significantly affected both the belowground and aboveground biomass of *Bt* rice (*P* < 0.05 or 0.001; Table 1). Both the belowground and aboveground biomass significantly increased with increased N-fertilizer augmentation, respectively (*P* < 0.05; Fig. 1). Compared with ambient CO_2_, elevated CO_2_ significantly increased the aboveground biomass of *Bt* rice grown at 2 N-fertilizer level (+ 25.74%), and belowground biomass of *Bt* rice grown at 1 N and 2 N-fertilizer levels (+ 27.71% and + 21.19%; *P* < 0.05, Fig. 1).

**Table 1:** Two-way ANOVAs for the effects of CO_2_ and N-fertilizer levels, and their interaction on the belowground and aboveground biomass, foliar contents of total soluble protein and Bt toxin, *Bt*-transgene expression and methylation in promoter and coding regions of *Bt* rice with fused *Cry1Ab/Ac* during tillering stage, grown under ambient and elevated CO_2_ with different N-fertilizer levels (*F* and *P* values).

Parameters	CO_2_ level (CO_2_)		N-fertilizer level (N)		CO_2_ × N	
	*F*-values	*P*-values	*F*-values	*P*-values	*F*-values	*P*-values
Aboveground biomass (g; fw)	9.30	0.003	193.81	< 0.001	3.29	0.04
Belowground biomass (g; fw)	12.24	< 0.001	244.28	< 0.001	3.25	0.04
Foliar total soluble protein (mg/g; fw)	1.43	0.24	15.73	< 0.001	2.02	0.15
Foliar Bt protein content (μg/g; fw)	0.58	0.46	72.99	< 0.001	3.54	0.045
*Bt* gene expression	3.84	0.07	4.55	0.03	16.61	< 0.001
Promoterregion methylation of P1 (%)	22.27	< 0.001	4.00	0.047*	23.54	< 0.001
Codingregion methylation of P2 (%)	1.61	0.23	0.05	0.95	3.02	0.086
Codingregion methylation of P3 (%)	0.004	0.95	0.28	0.76	0.46	0.64
Codingregion methylation of P2 + P3 (%)	1.70	0.22	0.13	0.88	3.92	0.049
Transgene methylation of P1 + P2 + P3 (%)	19.82	< 0.001	1.84	0.20	13.34	< 0.001

**Figure 2:**
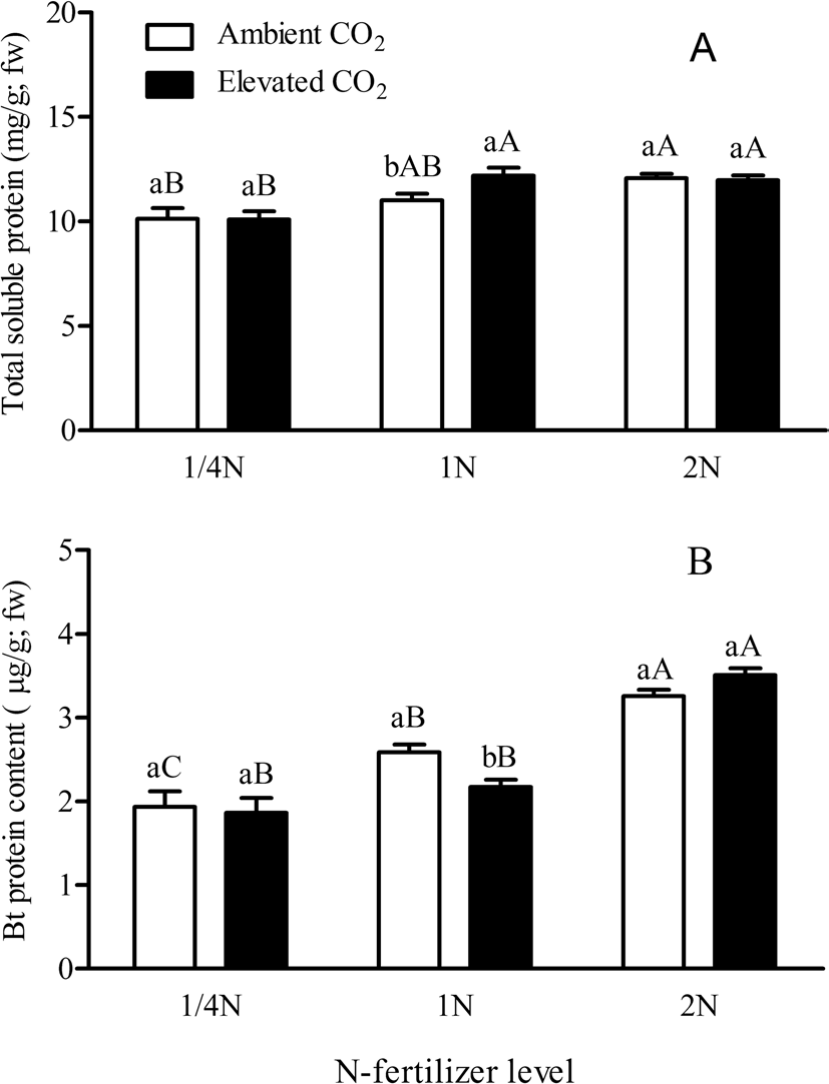
Foliar concentrations of total soluble protein (**A**) and Bt protein (**B**) in *Bt* rice with fused *Cry1Ab/Ac* during tillering stage, grown under ambient and elevated CO_2_ with different N-fertilizer level.

**Figure 3:**
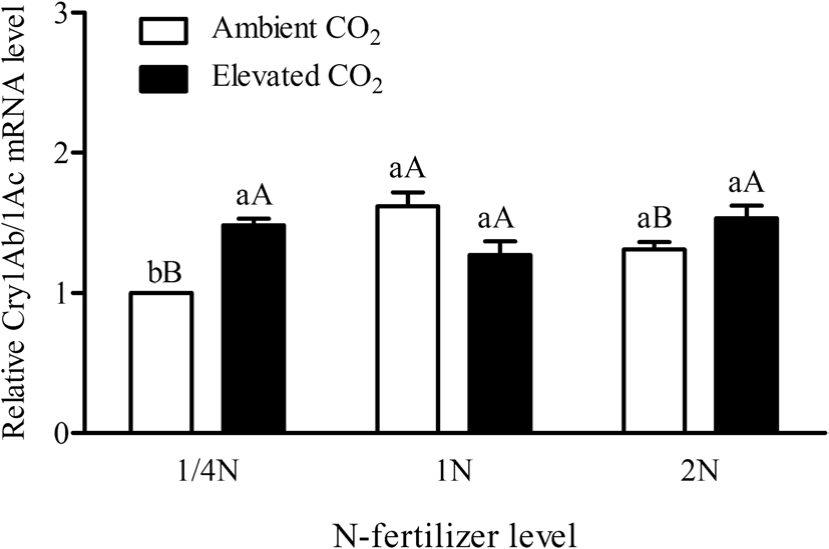
The relative transcript level of *Bt*-transgene in the leaves of *Bt* rice with fused *Cry1Ab/Ac* during tillering stage, grown under ambient and elevated CO_2_ with different N-fertilizer level.

**Figure 4:**
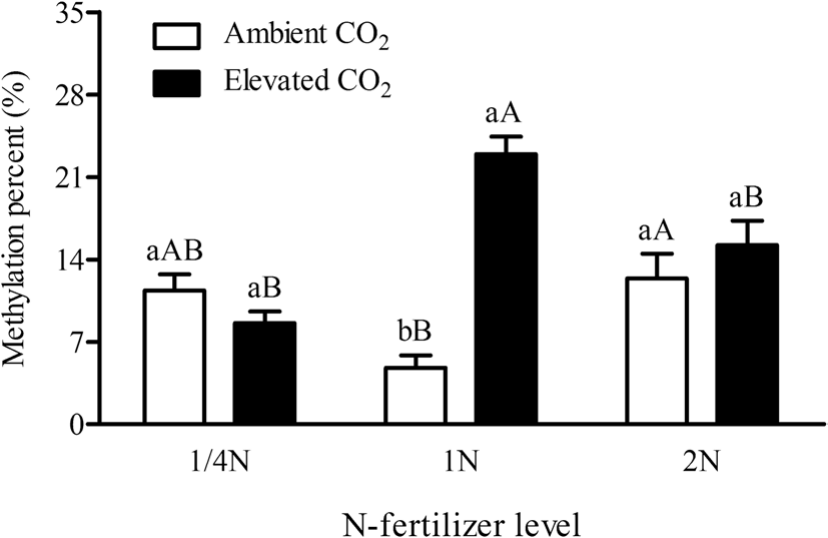
Cytosine methylation levels in the promoterregion (P1) of *Bt*-transgene in the leaves of *Bt* rice with fused *Cry1Ab/Ac* during tillering stage, grown under ambient and elevated CO_2_ with different N-fertilizer level.

**Figure5:**
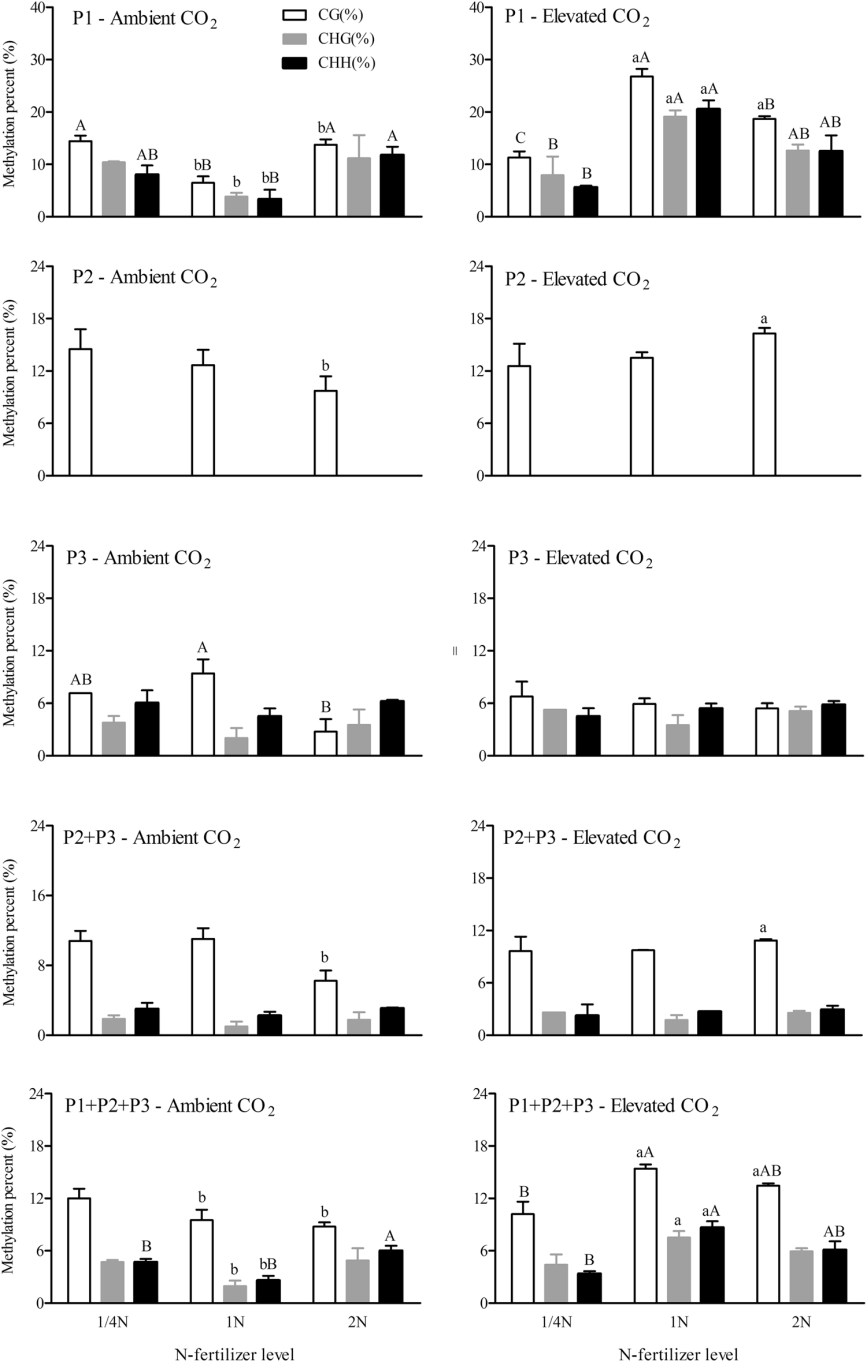
Percentage of different methylation patterns (CG, CHG and CHH) in the promoterregion (P1), and codingregion (P2, P3 and P2 + P3) of Bt-transgene (P1 + P2 + P3) in the leaves of *Bt* rice with fused *Cry1Ab/Ac* grown under ambient and elevated CO_2_ under three N-fertilizer levels.

**Figure 6:**
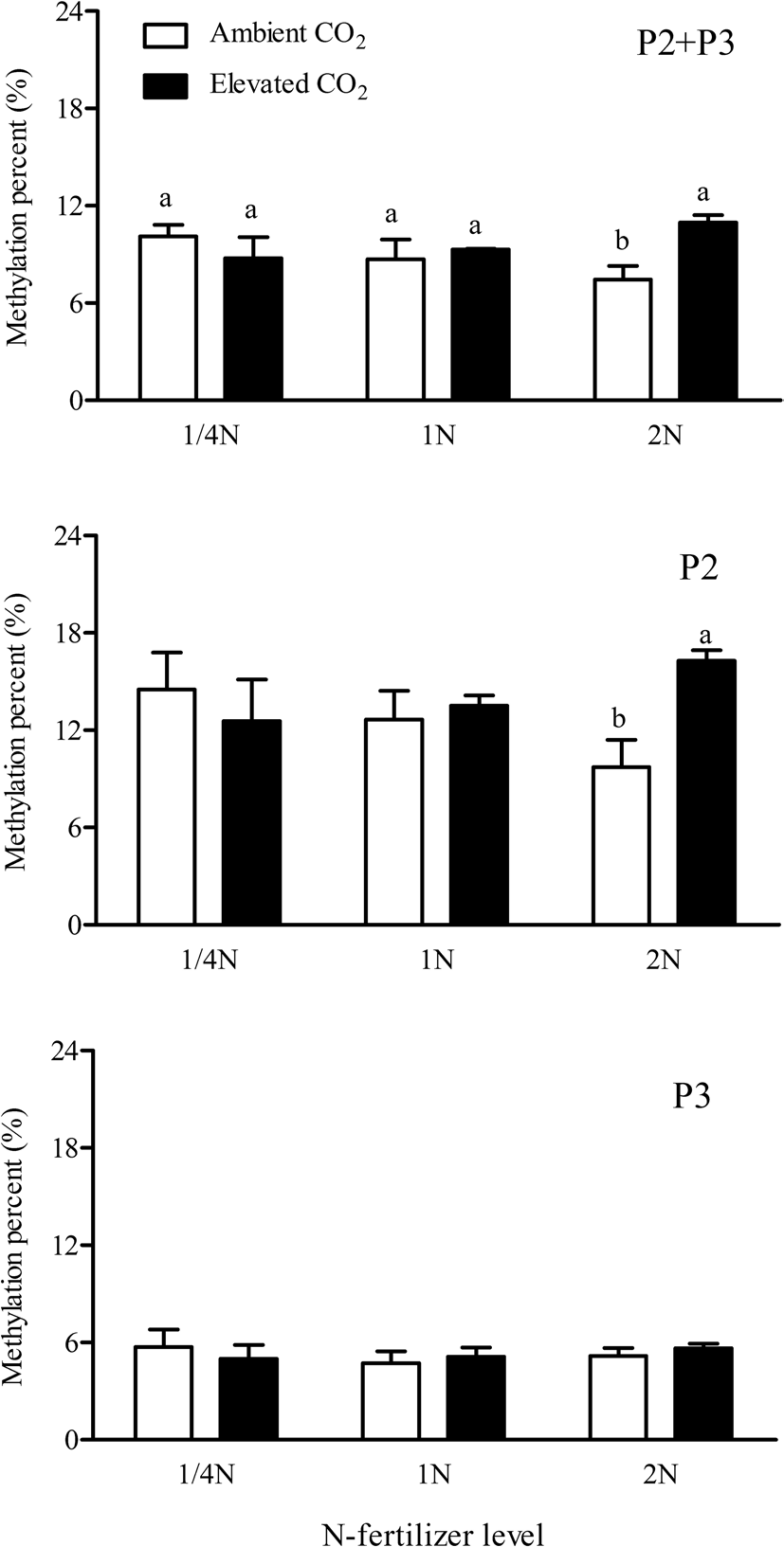
Cytosine methylation levels in the codingregion (P2, P3, P2 + P3) of Bt-transgene in the leaves of *Bt* rice with fused *Cry1Ab/Ac* during tillering stage, grown under ambient and elevated CO_2_ with different N-fertilizer level.

**Figure 7:**
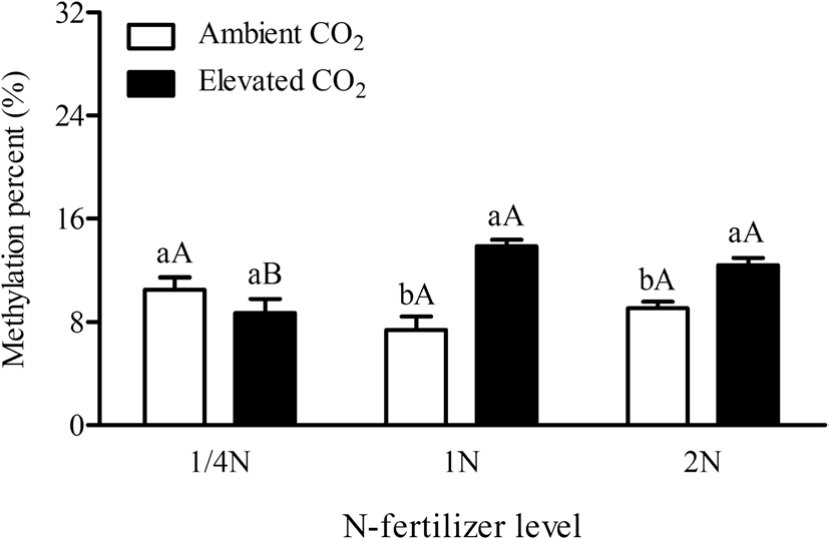
Cytosine methylation levels in the P1 + P2 + P3 of Bt-transgene in the leaves of Bt rice of the transgene promoter and coding-region in the leaves of transgenic *Bt* rice during tillering stage, grown under ambient and elevated CO_2_ with different N-fertilizer level.

**Figure 8:**
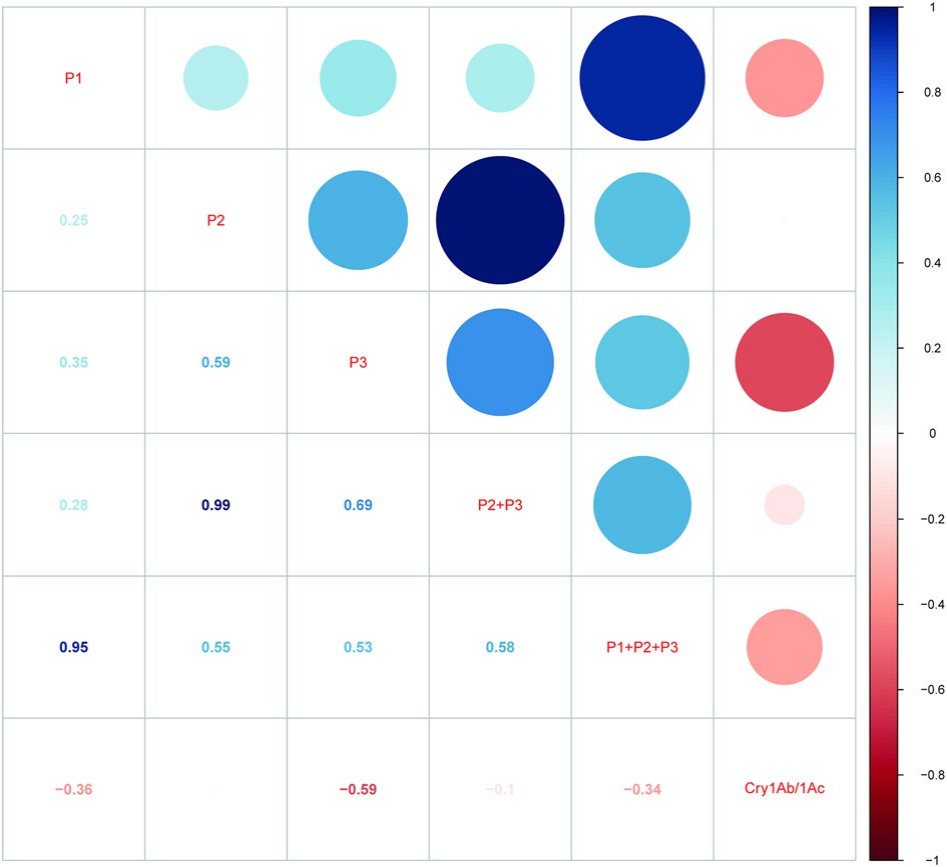
Pearson’s analysis on the correlations between the methylation levels in the promoterregion (P1), and codingregion (P2, P3, P2 + P3) of *Bt*-transgene (P1 + P2 + P3) and the *Cry1Ab/1Ac* expression level in the leaves of *Bt* rice leaves during tillering stage, grown under ambient and elevated CO2 with different N-fertilizer level. (P1, CpG island 1 (promoterregion); P2, CpG island 2 (codingregion); P3, CpG island 3 (codingregion); P2 + P3, CpG island 2 + CpG island 3 (codingregion); P1 + P2 + P3, CpG island 1 + CpG island 2 + CpG island 3 (*Bt*-transgene)).

### Foliar contents of total soluble protein and Bt protein of Bt rice

N-fertilizer level significantly affected the foliar content of total soluble protein of *Bt* rice (*P* < 0.001; Table 1). Under ambient CO_2_, the foliar content of total soluble proteins of *Bt* rice grown at 1/4 N level were significantly lower (− 16.14%) than that at 2 N level (*P* < 0.05; Fig. 2A). Under elevated CO_2_, the foliar content of total soluble proteins of *Bt* rice grown at reduced N-fertilizer level (1/4 N) were significantly lower than that at 1 N and 2 N levels (− 17.27% and − 15.70%; *P* < 0.05, Fig. 2A). Compared with ambient CO_2_, elevated CO_2_ significantly increased the foliar content of total soluble proteins of *Bt* rice grown at 1 N level (+ 10.75%; *P* < 0.05, Fig. 2A).

N-fertilizer level (*P* < 0.001) and its interaction with CO_2_ level (*P* < 0.05) significantly influenced the foliar *Bt* protein content of *Bt* rice (Table 1). Under ambient CO_2_, the foliar *Bt* protein content of *Bt* rice significantly increased with the N fertilizer augmentation (*P* < 0.05; Fig. 2B). Under elevated CO_2_, the foliar *Bt* protein content of *Bt* rice grown at 2 N level was significantly higher than that at 1/4 and 1 N levels (+ 88.21% and + 61.47%; *P* < 0.05; Fig. 2B). Compared with ambient CO_2_, elevated CO_2_ significantly decreased the foliar *Bt* protein content of *Bt* rice grown at 1 N level (− 16.04%; *P* < 0.05; Fig. 2B).

### Bt transgene expression in the leaves of Bt rice

N-fertilizer level (*P* < 0.05) and its interaction with CO_2_ level (*P* < 0.001) significantly affected the *Bt* transgene expression in the leaves of *Bt* rice (Table 1). Under ambient CO_2_, the *Bt*-transgene expression level in the leaves of *Bt* rice grown at 1/4 N and 2 N level was significantly down-regulated when compared with that at 1 N level (− 38.16% and − 19.04%; *P* < 0.05; Fig. 3). Compared with ambient CO_2_, elevated CO_2_ just significantly up-regulated the *Bt*-transgene expression level in the leaves of *Bt* rice grown at 1/4 N level (+ 48.03%; *P* < 0.05; Fig. 3).

### Methylation status in the promoterregion and codingregion of Bt-transgene in the leaves of Bt rice

#### Promoterregion (P1) of Bt-transgene

CO_2_, N-fertilizer levels and their interaction significantly affected the methylation levels in the promoterregion (P1) of *Bt*-transgene in the leaves of *Bt* rice (*P* < 0.05; Table1). N-fertilizer level differently affected the methylation in the P1 fragment of *Bt*-transgene in the leaves of *Bt* rice. The methylation percentages in the P1 fragment of *Bt*-transgene in the leaves of *Bt* rice grown at 1/4 N level (+ 135.89%) and 2 N level (+ 157.23%) were markedly higher than that at 1 N level under ambient CO_2_, respectively (*P* < 0.05; Fig. 4), while it was contrary tendency under elevated CO_2_. Significant decreases in the methylation percentages were found in the P1 fragment of *Bt*-transgene in the leaves of *Bt* rice grown at 1/4 N level (− 62.52%) and 2 N level (− 33.75%) in contrast to that at 1 N level under elevated CO_2_ (*P* < 0.05; Fig. 4). In addition, compared with ambient CO_2_, elevated CO_2_ obviously decreased the methylation percentages in the P1 fragment of *Bt*-transgene in the leaves of *Bt* rice grown at reduced N-fertilizer level (1/4 N) (− 24.21%; *P* > 0.05), and markedly enhanced the methylation percentages in the P1 fragment of *Bt*-transgene in the leaves of *Bt* rice grown at recommended normal (1 N: + 376.96%; *P* < 0.05) and increased N-fertilizer level (2 N: + 22.84%; *P* > 0.05, Fig. 4).

CO_2_, N-fertilizer levels and CO_2_ × N-fertilizer interactions significantly affected the methylation levels of cytosines located at CG and CHH sites in the P1 fragment of *Bt*-transgene in the leaves of *Bt* rice (*P* < 0.05; Table 2). The methylation levels of cytosines located at CHG site in the P1 fragment of *Bt*-transgene in the leaves of *Bt* rice was just significantly affected by CO_2_ and CO_2_ × N interactions (*P* < 0.05; Table 2). Under ambient CO_2_, the methylation level of cytosines located at CG and CHH sites in the P1 fragment of *Bt*-transgene in the leaves of *Bt* rice grown at 1/4 N level (+ 122.95% and + 140.32%; *P* < 0.05) and 2 N level (+ 112.82% and + 249.95%; *P* < 0.05) were markedly higher than that at 1 N level. In contrast, the methylation level of cytosines located at CG, CHG and CHH sites in the P1 fragment of *Bt*-transgene in the leaves of *Bt* rice grown at 1/4 N level (− 57.77%, − 58.41 and − 72.66%; *P* < 0.05) were significantly lower than that at 1 N level under elevated CO_2_ (Fig. 5). Moreover, compared with ambient CO_2_, elevated CO_2_ markedly enhanced the methylation percentages of cytosines located at CG, CHG and CHH sites in the P1 fragment of *Bt*-transgene in the leaves of *Bt* rice grown at 1 N level (+ 313.79%, + 397.40% and + 511.32%; *P* < 0.05), and CG sites in the P1 fragment of *Bt*-transgene in the leaves of *Bt* rice grown at increased N-fertilizer level (2 N: + 35.67%; *P* < 0.05) (Fig. 5). The methylation level of cytosines located at CG sites was higher than those at CHG and CHH in the P1 fragment of *Bt*-transgene in the leaves of *Bt* rice regardless of the CO_2_ or N-fertilizer level (Fig. 5).

**Table 2:** Two-way ANOVAs for the effects of CO_2_ and N-fertilizer levels, and their interaction on the cytosine methylation percentage in the promoterregion (P1) and codingregion (P2, P3, P2 + P3) of *Bt*-transgene (P1 + P2 + P3) in the leaves of *Bt* rice with fused *Cry1Ab/Ac* during tillering stage, grown under ambient and elevated CO_2_ with different N-fertilizer levels (*F* and *P* values).

Transgene region	Cytosine methylation patterns	CO_2_ level (CO_2_)		N-fertilizer level (N)		CO_2_ × N	
		*F*-values	*P*-values	*F*-values	*P*-values	*F*-values	*P*-values
Promoter region (P1)	CG (%)	66.98	< 0.001	7.01	0.0096	58.31	< 0.001
	CHG (%)	5.76	0.034	0.74	0.50	7.33	0.008
	CHH (%)	11.11	0.006	5.02	0.03	15.49	< 0.001
Codingregion (P2)	CG (%)	1.61	0.23	0.05	0.95	3.02	0.086
	CHG (%)	–	–	–	–	–	–
	CHH (%)	–	–	–	–	–	–
Codingregion (P3)	CG (%)	0.18	0.68	5.15	0.02	3.37	0.07
	CHG (%)	3.08	0.11	1.68	0.23	0.002	0.99
	CHH (%)	0.19	0.67	0.83	0.46	0.99	0.40
Codingregion (P2 + P3)	CG (%)	0.66	0.43	1.79	0.21	4.93	0.03
	CHG (%)	3.08	0.11	1.68	0.23	0.002	0.99
	CHH (%)	0.20	0.67	0.83	0.46	0.99	0.40
Transgene (P1 + P2 + P3)	CG (%)	18.64	0.001	2.24	0.15	6.72	0.01
	CHG (%)	18.38	0.001	3.63	0.06	3.57	0.06
	CHH (%)	15.47	0.002	10.41	0.002	12.12	0.001

#### Codingregion (P2, P3, P2 + P3) of Bt-transgene

The interaction between CO_2_ and N-fertilizer levels significantly affected the methylation levels in the codingregion (P2 + P3) of *Bt*-transgene in the leaves of *Bt* rice (*P* < 0.05; Table 1). Compared with ambient CO_2_, elevated CO_2_ significantly enhanced the methylation percentages in the P2 + P3 fragments of *Bt*-transgene in the leaves of *Bt* rice grown at increased N-fertilizer level (2 N: + 47.24%; *P* < 0.05) (Fig. 6). CO_2_ × N-fertilizer interaction significantly affected the methylation levels of cytosines located at CG site in the P2 + P3 fragments of *Bt*-transgenein the leaves of *Bt* rice (*P* < 0.05; Table 2). Compared with ambient CO_2_, elevated CO_2_ significantly enhanced the methylation percentages of cytosines located at CG sites in the P2 + P3 fragments of *Bt*-transgene in the leaves of *Bt* ricegrown at 2 N level (+ 67.52%; *P* < 0.05; Fig. 5). The methylation level of cytosines located at CG sites was higher than those at CHG and CHH sites in the P2 + P3 fragments of *Bt*-transgene in the leaves of *Bt* rice regardless of the CO_2_ or N-fertilizer level (Fig. 5).

CO_2_, Nitrogen-fertilizer levels and their interaction did not significantly affect methylation levels in the codingregion (P2) and codingregion (P3) of *Bt*-transgene in the leaves of *Bt* rice (*P* > 0.05; Table 1). In the codingregion (P2), the methylation percentage at 2 N level under elevated CO_2_ (16.28%) was significantly higher than that under ambient CO_2_ (9.72%) (*P* < 0.05, Fig. 6). There were no CHG and CHH sites as potential targets in the P2 fragment of *Bt*-transgene (Fig. 5). In the codingregion (P3), the methylated level was very low, not exceeding 5.74% (Fig. 6). The methylation level in the P3 fragment was lower than that in the P2 fragment of *Bt*-transgene in the leaves of *Bt* rice (Fig. 6). N-fertilizer level significantly influenced the methylation level of cytosines located at CG site in the P3 fragment of *Bt*-transgene in the leaves of *Bt* rice (*P* < 0.05; Table 2). Under ambient CO_2_, methylation level of cytosines located at CG sites in the P3 fragment of *Bt*-transgene in the leaves of *Bt* rice grown under increased N-fertilizer level (2 N) was significantly lower that at 1 N level (− 70.47%, *P* < 0.05; Fig. 5).

#### Bt-transgene (P1 + P2 + P3)

CO_2_ and its interaction with N-fertilizer significantly affected the methylation levels in the *Bt*-transgene (P1 + P2 + P3) in the leaves of *Bt* rice (*P* < 0.001; Table 1). The methylation percentages in the P1 + P2 + P3 fragments of *Bt*-transgene in the leaves of *Bt* rice grown at 1/4 N level were significantly lower than that at 1 N and 2 N level under elevated CO_2_ respectively (− 37.10% and − 15.80%; *P* < 0.05, Fig. 7). In addition, compared with ambient CO_2_, elevated CO_2_ markedly enhanced the methylation percentages in the P1 + P2 + P3 fragments of *Bt*-transgene in the leaves of *Bt* rice grown at recommended normal (1 N: + 87.17%; *P* < 0.05) and increased N-fertilizer level (2 N: + 36.17%; *P* > 0.05) (Fig. 7).

CO_2_, N-fertilizer levels and their interactions significantly affected the methylation levels of cytosines located at CHH sites in the P1 + P2 + P3 fragments of *Bt*-transgene in the leaves of *Bt* rice (*P* < 0.05; Table 2). The methylation levels of cytosines located at CG in the P1 + P2 + P3 fragments of *Bt*-transgene in the leaves of *Bt* rice was significantly affected by CO_2_ and its interaction with N-fertilizer (*P* < 0.05; Table 2), while the methylation levels of cytosines located at CHG sites in the P1 + P2 + P3 fragments of *Bt*-transgene in the leaves of *Bt* rice was just significantly affected by CO_2_ level. The methylation level of cytosines located at CG sites was higher than those at CHG and CHH sites in the P1 + P2 + P3 fragments of *Bt*-transgene in the leaves of *Bt* rice regardless of the CO_2_ or N-fertilizer level (Fig. 5). Under ambient CO_2_, the methylation level of cytosines located at CHH sites in the P1 + P2 + P3 fragments of *Bt*-transgene in the leaves of *Bt* rice grown at 2 N level were markedly higher than that at 1 N level (+ 128.29%; *P* < 0.05, Fig. 5). Under elevated CO_2_, the methylation level of cytosines located at CG and CHH sites in the P1 + P2 + P3 fragments of *Bt*-transgene in the leaves of *Bt* rice grown at 1/4 N level were significantly lower than that at 1 N level respectively (− 33.79% and − 61.01%; *P* < 0.05, Fig. 5). In addition, compared with ambient CO_2_, elevated CO_2_ markedly enhanced the methylation percentages of cytosines located at CG, CHG and CHH sites in the P1 + P2 + P3 fragments of *Bt*-transgene in the leaves of *Bt* rice grown at 1 N level (+ 62.03%, + 284.85% and + 229.98%; *P* < 0.05) and at CG sites in the P1 + P2 + P3 fragments of *Bt*-transgene in the leaves of *Bt* rice grown at increased N-fertilizer level (2 N: + 53.77%; *P* < 0.05) (Fig. 5).

### The correlation between the transgene methylation in promoterregion and codingregion, and the Bt-transgene expression level

The Pearson’s analysis showed that the methylation level in the promoterregion (P1) of *Bt*-transgene was negatively correlated with the *Cry1Ab/1Ac* expression level in the leaves of *Bt* rice (Fig. 8). The methylation level in the codingregion (P2 + P3) was slightly negatively correlated with the *Cry1Ab/1Ac* expression level in the leaves of *Bt* rice (Fig. 8). The methylation level in the *Bt*-transgene (P1 + P2 + P3) was negatively correlated with the *Cry1Ab/1Ac* expression level in leaves of *Bt* rice during tillering stage (Fig. 8).

## Discussion

Previous studies showed that elevated CO_2_ can stimulate plant growth and increase photosynthetic rate, photosynthate production, biomass and C: N ratios^36^. Hao et al. reported that the biomass of leaf, stem, pod, and total aboveground biomass of soybean increased with elevated CO_2_^37^. Our results indicated that elevated CO_2_ and increased N-fertilizer both increased the biomass of *Bt* rice. Also, it appeared that elevated CO_2_ showed a positive effect on the aboveground biomass of *Bt* rice grown under higher N-fertilizer (i.e., 2 N level) and belowground biomass of *Bt* rice grown under 1 N and 2 N-fertilizer. The biomass of *Bt* rice was significantly increased with increased augmentation of N fertilizer. It is expected that the increased nitrogen uptake by the plant would enhance the rate of photosynthesis, resulting in increased biomass accumulation via increased CO_2_ diffusion conductance and Rubisco content in *Bt* rice leaves^38–40^. Hence, elevated CO_2_ and augmentation of N supply simultaneously increased the rice biomass, likely manifesting synergistically additive effects on biomass accumulation.

In recent years, the potential impacts of future CO_2_ levels on *Bt* crops have attracted increasing attention. Our results show that foliar Bt protein content of *Bt* rice grown at elevated CO_2_ were significantly lower than that under ambient CO_2_ at 1 N level. It may be related to the decreased N allocation to Bt protein caused by elevated CO_2_^16^. Similarly, Coviella et al. found that elevated CO_2_ decreased Bt protein content in *Bt* cotton^22^. In this study, the foliar Bt toxin content of *Bt* rice at 2 N level was significantly higher than those at 1 N and 1/4 N level, indicating that the doubling of nitrogen augmentation (i.e., 2 N) resulted in the enhanced foliar Bt protein content level in the leaves of *Bt* rice. Bruns and Abel reported that the Bt protein production of two transgenic *Bt*-transgenic maize lines increased with the augmentation of N fertilizer application^41^. Yang et al. found that the contents of Cry2A and Cry1C in *Bt* rice both increased in the tillering and milking stages with the higher N concentrations applied on rice^42^. Wang et al. documented that the Cry1Ab/1Ac content of *Bt*-SY63 at higher N fertilizer was significantly higher than that without N fertilizer treatment^43^. Moreover, the foliar content of total soluble protein at 1/4 N level was significantly lower than that at 1 N and 2 N level, respectively. The Bt protein content in plant tissues has been shown to significantly correlate with soluble protein and overall nitrogen content^41,44^. Hence, it is plausible to increase the Bt protein content in *Bt* crops by taking appropriate nitrogen management measures.

Epigenetic changes in DNA methylation can affect transgene expression for transgenic crops. DNA methylation occurs in codingregion has a more complex association with gene expression, whereas DNA methylation in promoterregion plays a vital role in transgene silencing^35^. For example, the resistance marker expression of transformed tobacco cultivars was rapidly lost and transgene expression were down-regulated, and hypermethylation within the 35S and NOS-promoters of these cultivars were found^45^. Additionally, environmental factors, such as drought and extreme temperature can potentially influence the methylation status^46–48^. In rice, 70% of the drought-induced methylation changing sites were reversed to their original status after water recovery^49^. In this study, our results showed that elevated CO_2_ significantly enhanced the methylation percentages in the promoterregion (P1), and the P1 + P2 + P3 fragments of *Bt*-transgene in the leaves of *Bt* rice during tillering stage grown at 1 N level. In the codingregion, the methylation level in the P2 fragment of *Bt*-transgene, the fagment near the top strand of *Bt*-transgene, was higher than that in the P3 fragment, the fragment amplified from the bottom strand of *Bt*-transgene. Though the methylation level was low in P3 fragment of *Bt*-transgene, it was negatively correlated with the *Cry1Ab/1Ac* expressi in the leaves of *Bt* rice during tillering stage. In general, the methylation status in codingregion in *Bt*-transgene was slightly negatively correlated with the *Cry1Ab/1Ac* expression level in the leaves of *Bt* rice during tillering tage. Jiang et al. found that the PTGS methylation in the codingregion of *Bt*-transgene in the leaves of *Bt* rice during seeding stage remained at a relatively low level, lower than 5%^19^. The methylation level in the codingregion of *Bt*-transgene shows a weak regulation to the transgene expression. Thus, the methylation level in codingregionof *Bt*-transgene in the leaves of *Bt* rice has a weak regulation to the transgene expression both in tillering and seeding stage. The methylation levels in the promoterregion likely affected transgene expression more than that in the codingregion of *Bt*-transgene in the leaves of *Bt* rice. In addition, the Pearson’s analysis also showed that the methylation level in the P1 + P2 + P3 fragments of *Bt*-transgene was negatively correlated with the *Cry1Ab/1Ac* expression in the leaves of *Bt* rice. Thus, the methylation level in the P1 + P2 + P3 fragments of *Bt*-transgene was showed moderate regulation to the transgene expression in the leaves of *Bt* rice during tillering stage.

Stable transgene expression and heritability are key factors for the development and application of transgenic crops. Environmental factors, such as soil salinity, water accessibility and temperature all play crucial roles in *Bt* transgene expression^50,51^. Trtikova et al. found that the *Cry1Ab* expression in MON 810 maize under hot/dry stress was significantly lower than that under optimal conditions^52^. Other studies with *Bt* crops have also indicated that environment might influence the levels of transgene expression differently^53^. Our results indicated that the *Bt* transgene expression was significantly up-regulated by elevated CO_2_ under 1/4 N level, and *Bt* transgene expression level in the leaves of Bt rice grown at 1/4N and 2N level was significantly down-regulated when compared with that at 1N level under ambient CO_2_. Considering the methylation level in promoterregion and codingregion of *Bt*-transgene was negatively correlated with the *Cry1Ab/1Ac* expression level in the leaves of *Bt* rice during tillerage stage, so we speculate that the different transgene expression level among different CO_2_ and N treatments was caused by methylation in promoterregion and codingregion of *Bt*-transgene and post-transcriptional regulation in the leaves of *B*t rice during tillering stage.

In conclusion, the methylation level in the promoterregion and codingregion of *Bt*-transgene were negatively correlated with the *Bt* transgene expression level in the leaves of *Bt* rice during tillering stage. The methylation levels in the promoterregion likely affected transgene expression more than that in the codingregion of *Bt*-transgene in the leaves of *Bt* rice during tillering stage. Elevated CO_2_ showed positively effect on the transgene methylation level and negatively effect on the foliar *Bt* toxin content of *Bt* rice grown under 1 N level. The increased N-fertilizer level showed positively effect on the foliar *Bt* toxin content of *Bt* rice during tillering stage. Under elevated CO_2_ situation in the future, moderate application of N-fertilizer can increase the foliar Bt toxin content in *Bt* rice. Futhermore, additional studies should be performed to evaluate the efficacy of the transgenic proteins against the target organisms under elevated CO_2_, and thus the biological meaning behind it.

## Materials and methods

### Plant materials

**Figure 9:**
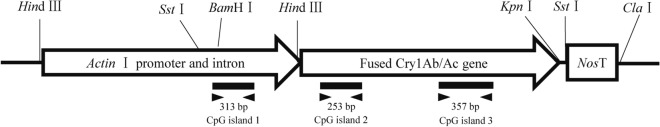
Schematic diagram of the fused *Cry1Ab/Ac* gene and its plasmid.

The *Bt* rice cultivar HH1 (Huahui 1) was used in the study. The rice seeds were provided by Prof. Yongjun Lin from Huazhong Agricultural University (Wuhan, China). HH1 was developed by using MH63 as the recipient to harbor the fusion gene *Cry1Ab*/*Ac* from transgenic event TT51-1 (GenBank Accession Number: EU880444.1). Expression of the *Cry1Ab*/*Ac* gene is driven by the rice *actin 1* promoter and the nopaline synthase (NOS) gene terminator (seen in Fig. 9).

### Plant growth conditions

This experiment was performed in electronically controlled growth incubator (GDN-400D-4/CO_2_; Ningbo Southeast Instrument CO., LTD, Ningbo, China) connected with a gas-tank system for maintaining the desired atmospheric CO_2_ concentration. The conditions in the chambers were maintained at 28 °C (day) and 25 °C (night) under a 16: 8 h light/dark photoperiod. The light intensity was 20,000 lx. Two CO_2_ concentrations levels were applied continuously, i.e., elevated CO_2_ (800 ppm, predicted CO_2_ concentration in 2100), and ambient CO_2_ (about 400 ppm). With each CO_2_ level, the N-fertilizer was set at three levels, 1/4, 1 and 2 N; the 1 N was 1.25 mM NH_4_NO_3_. Therefore, the experiment was consisted of 2 CO_2_ concentrations × 3 N-fertilizer levels (total 6 treatment combinations) deployed in six electronically controlled growth incubators as three replications for CO_2_ main factors.

The rice seeds of *Bt* rice (cv. HH1) were soaked in water for one day, and germinated on a board covered with wet cotton gauze for one day. Then, these seeds were sown into plastic foam covering (0.6 cm thick) on plastic cups (9 cm diameter, 7 cm height) and placed in the electronically controlled growth incubators of ambient and elevated CO_2_. In the cup, there were two holes in the plastic foam and one rice seeds into each hole (total two seeds per cup). Thirty cups were placed in each electronically controlled growth chambers with 10 cups per N-fertilizer level. The cups were filled with modified culture solutions^54^; the solution was replaced with fresh solution every day. The composition of modified culture solutions was as follows (per liter): NH_4_NO_3_, 1.25 mM; KH_2_PO_4_, 0.3 mM; K_2_SO_4_, 1 mM; CaCl_2_·2H_2_O, 1 mM; MgSO_4_·7H_2_O, 1 mM; Na_2_SiO_3_·9H_2_O, 0.5 mM. (2) Micronutrient solution: MnCl_2_·4H_2_O, 9 μM; Na_2_MoO_4_·2H_2_O, 0.39 μM; H_3_BO_3_, 20 μM; ZnSO_4_·7H_2_O, 0.77 μM; CuSO_4_·5H_2_O, 0.32 μM; FeSO_4_·7H_2_O + Na_2_-EDTAN^54^. The plastic cups (plants) were re-randomized every two days within the chamber to minimize the positional effect. At tillering stage, the rice plants were collected, labelled, and stored at − 80 °C for various measurements.

### Measurement of plant biomass

After sixty-five days for *Bt* rice grown under ambient and elevated CO_2_ with different N-fertilizer levels (i.e., tillering stage), ten *Bt* rice plants for each N-fertilizer level were randomly selected from each growth incubator (i.e., 30 rice plants for each fertility-fertilizer level per CO_2_ level). The biomass of belowground (root) and aboveground (stem and leaves) plant tissues were individually weighted with an electronic balance (Mettler Toledo AL 104; readability = 0.1 mg, repeatability < ± 0.1 mg).

### Measurement of foliar contents of total soluble protein and Bt protein

After the measurement of plant biomass, the foliar contents of total soluble protein and *Bt* protein in the sampled rice plants were measured using the diagnostic kit, A045-2 (Nanjing Jiancheng Bioengineering Institute) and ELISA kits from EnviroLogix (Portland, ME; catalog number AP003), respectively. Three leaves from each sampled plant were taken as a sample unit and weighed. Five samples were measured for each treatment. The samples were individually placed into 2 ml microreaction tubes and homogenized in a Tissue Lyser II (Qiagen) by shaking for 3 min at 30 Hz with two steel balls in each tube. For the determination of foliar total soluble protein content, 0.9% saline was used as an extraction buffer in a proportion of 1:9 (m/v). Then, the measurement was performed by following the kit instructions. Optical density (OD) values were measured using a UV–Vis spectrophotometer (UV-1800PC, Mapada, Shanghai, China) at 595 nm wavelength. For the determination of foliar *Bt* protein content, samples were mixed with extraction buffer PBST (provided with the kit) in a proportion of 1: 10 to 1: 100 (m/v) and then measured the foliar *Bt* protein content in the leaves of *Bt* rice during tillering stage according to the kit instructions. The OD values were measured using a UV–Vis spectrophotometer at 450 nm wavelength.

### Bioassay of the transcript expression levels of Bt-transgene

#### RNA extraction and reverse transcription

One leaf per rice plant was excised from 3 plants (total 3 leaves per replication) of each treatment combination of CO_2_ and N-fertilizer levels for quantification of transcript expression levels of *Bt*-transgene in the leaves of *Bt* rice during tillering stage. Three samples were measured for each treatment. Total RNA was extracted from leaf tissues using TRIzol reagent following the supplier’s protocol (Invitrogen). RNA concentration and integrity were evaluated using the NanoDrop spectrophotometer (Thermo Scientific). First strand cDNA templates were synthesized using Prime Script RT reagent kit (TaKaRa, Japan).

#### Real-time PCR analysis

Quantitative real-time PCR (qRT-PCR) experiment was carried out using SYBR Premix Ex Taq (TaKaRa, Japan) following the kit instructions. Expression of the target gene (i.e., *Bt*-transgene) was normalized relative to the expression of the housekeeping genes actin1 and ubiquitin. Quantification of the transcript level of *Bt*-transgene in the leaves of *Bt* rice during tillering stage was based on the method of Livak and Schmittgen^55^. Primers used for qRT-PCR are listed in Table 3.

**Table 3:** Primers used for qRT-PCR in quantifying transcript expression levels of *Bt* transgene.

Primer	Sequence (5′-3′)	GeneBank accession	Description
Cry1Ab/Ac-F	TAGAGTTCGTGTGAGGTA	EU816953	*Bt* protein gene
Cry1Ab/Ac-R	CTGTATTGGAGAAGATGGAT		
Actin1-F	ATGGCAACATTGTGCTCAGTG	*Bt*130427^95^	Rice housekeeping gene
Actin1-R	CCTCCGATCCAGACGCTGTA		
Ubiquitin-F	GCTCCGTGGCGGTATCAT	NC_029258^96^	Rice housekeeping gene
Ubiquitin-R	CGGCAGTTGACAGCCCTAG		

### Methylation analysis of Bt-transgene

Genomic DNA were extracted and purified from 30 mg treated leaves of Bt rice from each treatment combination of CO_2_ and N-fertilizer levels during tillering stage using DNAsecure Plant Kit (TIANGEN, Beijing, China) following the product instructions. DNA concentration was quantified in the NanoDrop spectrophotometer. Then, 100 ng of isolated DNA was submitted to bisulfite treatment to convert non-methylated cytosines into uracil. The conversion was performed using the DNA Bisulfite Conversion Kit (TIANGEN, Beijing, China). Three types of cytosines -CG, CHG and CHH were analyzed in two regions of transgene: a fragment of the Actin 1 promoter (P1, CpG island 1) and two fragments of *Cry1Ab/1Ac* coding region (P2, CpG island 2 and P3,CpG island 3) (Table 4). The bisulfite sequencing primers were designed using Methyl Primer Express Software (Applied Biosystems) (Table 5).

**Table 4:** DNA sequences of CpG islands in the protmoterregion (P1) and coddingregion (P2 and P3) of *Bt*-transgene in the leaves of *Bt* rice during tillering stage, grown under ambient and elevated CO_2_ with different N-fefrtilizer levels.

CpG island	Sequence (5′–3′)
CpG island 1	TTTTTGGTTTTGGTAGTTTGGGTGGGCGAGAGGCGGCTTCGTGCGCGCCCAGATCGGTGCGCGGGAGGGGCGGGATCTCGCGGCTGGGGCTCTCGCCGGCGTGGATCCGGCCCGGATCTCGCGGGGAATGGGGCTCTCGGATGTAGATCTGCGATCCGCCGTTGTTGGGGGAGATGATGGGGGGTTTAAAATTTCCGCCATGCTAAACAAGATCAGGAAGAGGGGAAAAGGGCACTATGGTTTATATTTTTATATATTTCTGCTGCTTCGTCAGGCTTAGATGTGCTAGATTTTTTTTTTTTTTTTTTGTGGG
CpG island 2	TTGGTGTAAATTGAGTAGTTGATTAATTAGAGGATCGAAGAGTTCGTTAGGAATTAGGTTATTTTTAGGTTGGAAGGATTGAGTAATTTTTATTAAATTTATGTAGAGAGTTTTAGAGAGTGGGAAGTCGATTTTATTAATTAAGTTTTTCGCGAGGAAATGCGTATTTAATTTAACGATATGAATAGCGTTTTGATTATAGTTATTTTATTGTTCGTAGTTTAGAATTATTAAGTTTTTTTTTTGTTCGTGT
CpG island 3	GGAGAGTATTACTGGTCTGGACACCAGATCATGGCCTCTCCAGTTGGATTCAGCGGGCCCGAGTTTACCTTTCCTCTCTATGGAACTATGGGAAACGCCGCTCCACAACAACGTATCGTTGCTCAACTAGGTCAGGGTGTCTACAGAACCTTGTCTTCCACCTTGTACAGAAGACCCTTCAATATCGGTATCAACAACCAGCAACTTTCCGTTCTTGACGGAACAGAGTTCGCCTATGGAACCTCTTCTAACTTGCCATCCGCTGTTTACAGAAAGAGCGGAACCGTTGATTCCTTGGACGAAATCCCACCACAGAACAACAATGTGCCACCCAGGTAAGGATTTTTTTATAGGTTG

**Table 5:** Primers for bisulfite sequencing of *Bt*-transgene in the leaves of *Bt* rice during tillering stage, grown under ambient and elevated CO_2_ with different N-fefrtilizer levels.

Primer	Sequence (5′–3′)	Description
P1-F	TTTTTGGTTTTGGTAGTTTGG	CpG island 1
P1-R	CCCACAAAAAAAAAAAAAAAAA	
P2-F	TTGGTGTAAATTGAGTAGTTGAT	CpG island 2
P2-R	ACACRAACAAAAAAAAAACTTA	
P3-F	GGAGAGTATTATTGGTTTGGATA	CpG island 3
P3-R	CAACCTATAAAAAAATCCTTACCT	

The target sequences of *Bt*-transgene were amplified from the Bisulfite-treated genomic DNA by PCR with Methylation-specific Kit (TIANGEN, Beijing, China). The PCR conditions consisted of denaturation at 95 °C for 5 min, followed by 35 cycles at 94 °C for 20 s, 60 °C for 30 s, 72 °C for 20 s, and annealing at 72 °C for 5 min. The PCR products were purified using AxyPrep DNA Gel Extraction Kit (Axygen, Union City, USA), cloned into *pEASY*-T3 Cloning Vector and transformed into *Trans* 1-T1 Phage Resistant Chemically Competent Cell (TransGen, Beijing, China). Positive clones were screened with PCR using M13R and M13F primers. Sequencing were done for at least ten independent positive clones from each PCR product was carried out.

### Data analysis

All statistical analyses were conducted using SPSS (version 22.0; SPSS Inc., Chicago IL, USA; https://www.ibm.com/products/spss-statistics). DNA methylation levels (%) in CG, CHG and CHH cytosine types were assessed using the kismeth web tool. Two-way analysis of variances (ANOVAs) were performed to examine the effects of CO_2_ (Ambient vs. Elevated) and N-fertilizer (1/4, 1 and 2 N), and their interactions on plant biomass, foliar contents of total soluble protein and *Bt* protein, the gene expression levels of *Cry1Ab/Ac*, and the methylation level in the promoterregion (P1) and codingregion (P2, P3, P2 + P3) of *Bt-*transgene (P1 + P2 + P3) in the leaves of *Bt* rice during tillering stage. If there were significant effects of CO_2_ level, N-fertilizer level or their interaction, the least significant difference (LSD) test was used to separate the treatment means at *P* < 0.05. The Pearson’s test was performed by R software (version R i386 3.4.2; https://www.r-project.org/) to analyze correlations among methylation level in promoter region and coding region of *Bt*-transgene with the transgene expression level in the leaves of *Bt* rice during tillering stage, grown under ambient and elevated CO_2_ with different N-fertilizer levels.

## References

[1] Long SP, Ort DR (2010). More than taking the heat: Crops and global change. Curr. Opin. Plant Biol..

[2] IPCC (2014). Impacts, Adaptation and Vulnerability. Working Group II Contribution to the Fifth Assessment Report of the Intergovernmental Panel on Climate Change.

[3] Ainsworth EA, Rogers A (2007). The response of photosynthesis and stomatal conductance to rising CO2: Mechanisms and environmental interactions. Plant Cell Environ..

[4] Jackson RB, Cook CW, Pippen JS, Palmer SM (2009). Increased belowground biomass and soil CO_2_ fluxes after a decade of carbon dioxide enrichment in a warm-temperate forest. Ecology.

[5] Liu Y, Dang Z, Parajulee MN, Chen F (2019). Interactive effects of CO_2_ and temperature on plant chemistry of transgenic bt rice and population dynamics of a non-target planthopper, nilaparvata lugens (stal) under different levels of soil nitrogen. Toxins.

[6] Zavala JA, Nabity PD, DeLucia EH (2013). An emerging understanding of mechanisms governing insect herbivory under elevated CO_2_. Annu. Rev. Entomol..

[7] Hartley SE, Jones CG, Couper GC, Jones TH (2000). Biosynthesis of plant phenolic compounds in elevated atmospheric CO_2_. Glob. Change Biol..

[8] Bidart-Bouzat MG, Mithen R, Berenbaum MR (2005). Elevated CO_2_ influences herbivory-induced defense responses of *Arabidopsis thaliana*. Oecologia.

[9] Sun Y, Cao H, Yin J, Kang L, Ge F (2010). Elevated CO_2_ changes the interactions between nematode and tomato genotypes differing in the JA pathway. Plant Cell Environ..

[10] Xu HP, Xie HC, Wu SY, Wang ZY, He KL (2019). Effects of elevated CO_2_ and increased N fertilization on plant secondary metabolites and chewing insect fitness. Front. Plant Sci..

[11] Li Y, Hallerman EM, Liu Q, Wu K, Peng Y (2016). The development and status of Bt rice in China. Plant Biotechnol. J..

[12] Chen F, Wu G, Ge F, Parajulee MN (2011). Relationships between exogenous-toxin quantity and increased biomass of transgenic Bt crops under elevated carbon dioxide. Ecotoxicol. Environ. Saf..

[13] Li Y, Peng Y, Hallerman EM, Wu K (2014). Biosafety management and commercial use of genetically modified crops in China. Plant Cell Rep..

[14] Lu BR (2016). Challenges of transgenic crop commercialization in China. Nat. Plants.

[15] Wang YN (2016). Comparison of three transgenic Bt rice lines for insecticidal protein expression and resistance against a target pest, *Chilo suppressalis* (Lepidoptera: Crambidae). Insect Sci..

[16] Coviella CE, Stipanovic RD, Trumble JT (2002). Plant allocation to defensive compounds: interactions between elevated CO_2_ and nitrogen in transgenic cotton plants. J. Exp. Bot..

[17] Chen FJ, Wu G, Ge F, Parajulee MN, Shrestha RB (2005). Effects of elevated CO_2_ and transgenic Bt cotton on plant chemistry, performance, and feeding of an insect herbivore, the cotton bollworm. Entomol. Exp. Appl..

[18] Chen M, Shelton A, Ye GY (2011). Insect-Resistant genetically modified rice in China: From research to commercialization. Annu. Rev. Entomol..

[19] Jiang S (2017). Impacts of elevated CO_2_ on exogenous *Bacillus thuringiensis* toxins and transgene expression in transgenic rice under different levels of nitrogen. Sci. Rep..

[20] Himanen SJ (2008). Interactions of elevated carbon dioxide and temperature with aphid feeding on transgenic oilseed rape: Are *Bacillus thuringiensis* (Bt) plants more susceptible to nontarget herbivores in future climate?. Glob. Change Biol..

[21] Tsutsumi K, Konno M, Miyazawa SI, Miyao M (2014). Sites of action of elevated CO_2_ on leaf development in rice: Discrimination between the effects of elevated CO_2_ and nitrogen deficiency. Plant Cell Physiol..

[22] Coviella CE, Trumble JT (2000). Effect of elevated atmospheric carbon dioxide on the use of foliar application of Bacillus thuringiensis. Biocontrol.

[23] Hu LF (2011). Rice MADS3 regulates ROS homeostasis during late anther development. Plant cell.

[24] Jullien PE, Susaki D, Yelagandula R, Higashiyama T, Berger F (2012). DNA methylation dynamics during sexual reproduction in *Arabidopsis thaliana*. Curr. Biol..

[25] Ma Y (2018). Disrupted genome methylation in response to high temperature has distinct affects on microspore abortion and anther indehiscence. Plant Cell.

[26] Matzke MA, Mosher RA (2014). RNA-directed DNA methylation: An epigenetic pathway of increasing complexity. Nat. Rev. Genet..

[27] Zhong S (2013). Single-base resolution methylomes of tomato fruit development reveal epigenome modifications associated with ripening. Nat. Biotechnol..

[28] Yong-Villalobos L (2015). Methylome analysis reveals an important role for epigenetic changes in the regulation of the Arabidopsis response to phosphate starvation. Proc. Natl. Acad. Sci. U.S.A..

[29] Mette MF (2000). Transcriptional silencing and promoter methylation triggered by double-stranded RNA. Embo J..

[30] Matzke M (2004). Genetic analysis of RNA-mediated transcriptional gene silencing. Biochim. Biophys. Acta.

[31] Matzke M, Kanno T, Huettel B, Daxinger L, Matzke AJM (2007). Targets of RNA-directed DNA methylation. Curr. Opin. Plant Biol..

[32] Dalakouras A, Dadami E, Zwiebel M, Krczal G, Wassenegger M (2012). Transgenerational maintenance of transgene body CG but not CHG and CHH methylation. Epigenetics.

[33] Lister R (2008). Highly integrated single-base resolution maps of the epigenome in Arabidopsis. Cell.

[34] Vermeersch L (2013). Transitive RNA silencing signals induce cytosine methylation of a transgenic but not an endogenous target. Plant J..

[35] Li MY (2013). NaCl-induced changes of ion fluxes in roots of transgenic *Bacillus thuringiensis* (Bt) cotton (*Gossypium hirsutum* L.). J. Integr. Agric..

[36] Drake BG, Gonzalez-Meler MA, Long SP (1997). More efficient plants: A consequence of rising atmospheric CO_2_?. Annu. Rev. Plant Biol..

[37] Hao XY (2009). Effects of free air CO_2_ enrichment (FACE) on growth and yield of summer soybean. Acta Ecol. Sin..

[38] Yamori W, Nagai T, Makino A (2011). The rate-limiting step for CO_2_ assimilation at different temperatures is influenced by the leaf nitrogen content in several C-3 crop species. Plant Cell Environ..

[39] Reich PB, Hobbie SE, Lee TD (2014). Plant growth enhancement by elevated CO_2_ eliminated by joint water and nitrogen limitation. Nat. Geosci..

[40] Ruiz C, Pla M, Company N, Riudavets J, Nadal A (2016). High CO_2_ concentration as an inductor agent to drive production of recombinant phytotoxic antimicrobial peptides in plant biofactories. Plant Mol. Biol..

[41] Bruns HA, Abel CA (2003). Nitrogen fertility effects on Bt delta-endotoxin and nitrogen concentrations of maize during-early growth. Agron. J..

[42] Yang Y (2016). Impacts of nitrogen fertilizer on major insect pests and their predators in transgenic Bt rice lines T2A–1 and T1C–19. Entomol. Exp. Appl..

[43] Wang F (2012). Effects of N treatments on the yield advantage of Bt-SY63 over SY63 (*Oryza sativa*) and the concentration of Bt protein. Field Crop. Res..

[44] Dong HZ, Li WJ (2007). Variability of endotoxin expression in Bt transgenic cotton. J. Agron. Crop Sci..

[45] Weinhold A, Kallenbach M, Baldwin IT (2013). Progressive 35S promoter methylation increases rapidly during vegetative development in transgenic *Nicotiana attenuata* plants. Bmc Plant Biol..

[46] Fan HH (2013). DNA methylation alterations of upland cotton (*Gossypium hirsutum*) in response to cold stress. Acta Physiol. Plant..

[47] Xia H (2017). Differentially methylated epiloci generated from numerous genotypes of contrasting tolerances are associated with osmotic-tolerance in rice seedlings. Front. Plant Sci..

[48] Chen B, Saltveit ME, Beckles DM (2019). Chilling-stress modifies DNA methylation level in cucumber (*Cucumis sativus* L.) seedling radicle to regulate elongation rate. Sci. Hortic..

[49] Wang W (2011). Drought-induced site-specific DNA methylation and its association with drought tolerance in rice (*Oryza sativa* L.). J. Exp. Bot..

[50] Stam M, Mol JNM, Kooter JM (1997). The silence of genes in transgenic plants. Ann. Bot..

[51] Vilperte V, Agapito-Tenfen SZ, Wikmark OG, Nodari RO (2016). Levels of DNA methylation and transcript accumulation in leaves of transgenic maize varieties. Environ. Sci. Eur..

[52] Trtikova M, Wikmark OG, Zemp N, Widmer A, Hilbeck A (2015). Transgene expression and bt protein content in transgenic Bt maize (MON810) under optimal and stressful environmental conditions. PLoS ONE.

[53] Xia LQ, Guo SD (2004). The expression of Bt toxin gene under different thermal treatments. Sci. Agric. Sin..

[54] Kumar A, Silim SN, Okamoto M, Siddiqi MY, Glass ADM (2003). Differential expression of three members of the AMT1 gene family encoding putative high-affinity NH_4_+ transporters in roots of *Oryza sativa* subspecies indica. Plant Cell Environ..

[55] Livak K. J. & Schmittgen T. D. Analysis of relative gene expression data using real-time quantitative PCR and the 2^−ΔΔCT^ method. *Methods*. **25**, 402–408 (2001). 10.1006/meth.2001.1262 11846609

